# Recipient design in human communication: simple heuristics or perspective taking?

**DOI:** 10.3389/fnhum.2012.00253

**Published:** 2012-09-25

**Authors:** Mark Blokpoel, Marlieke van Kesteren, Arjen Stolk, Pim Haselager, Ivan Toni, Iris van Rooij

**Affiliations:** Radboud University Nijmegen, Donders Institute for Brain Cognition and BehaviourNijmegen, Netherlands

**Keywords:** heuristics, recipient design, communication, computational intractability

## Abstract

Humans have a remarkable capacity for tuning their communicative behaviors to different addressees, a phenomenon also known as *recipient design*. It remains unclear how this tuning of communicative behavior is implemented during live human interactions. Classical theories of communication postulate that recipient design involves *perspective taking*, i.e., the communicator selects her behavior based on her hypotheses about beliefs and knowledge of the recipient. More recently, researchers have argued that perspective taking is computationally too costly to be a plausible mechanism in everyday human communication. These researchers propose that computationally simple mechanisms, or *heuristics*, are exploited to perform recipient design. Such heuristics may be able to adapt communicative behavior to an addressee with no consideration for the addressee's beliefs and knowledge. To test whether the simpler of the two mechanisms is sufficient for explaining the “how” of recipient design we studied communicators' behaviors in the context of a non-verbal communicative task (the Tacit Communication Game, TCG). We found that the specificity of the observed trial-by-trial adjustments made by communicators is parsimoniously explained by perspective taking, but not by simple heuristics. This finding is important as it suggests that humans do have a computationally efficient way of taking beliefs and knowledge of a recipient into account.

## 1. Introduction

Imagine that a person on the street comes up to Ann and asks her: “Where can I find a supermarket?” Ann's reply may depend in subtle ways on a multiplicity of cues such as whether or not the person speaks with a foreign accent, the person is speaking hastily, or the person is by car. In the presence of such cues she may, for instance, speak more clearly, use simpler words, make shorter sentences, and give directions specifically how to drive there by car. As a result of these adjustments Ann may construct a message that the addressee is more likely to understand than otherwise. This adaptation of a communicative signal—such that it is tuned to the addressee—is known as *recipient design* (Sacks et al., 1974).

Classical theories of communication consider recipient design as constitutive of genuine or intentional communication (Grice, 1975, 1989; Levelt, 1989), as opposed to mere accidental or non-intentional forms of communication. Yet, recently a debate has ensued on the presumed ubiquity of recipient design in everyday communication (Clark, 1996; Horton and Keysar, 1996; Keysar et al., 1998), as well as on the nature of the cognitive mechanisms underlying the phenomenon (Epley et al., 2004; Shintel and Keysar, 2009; Galati and Brennan, 2010). With this paper we aim to contribute particularly to the second topic of debate: i.e., the nature of the mechanisms underlying recipient design in everyday (interactive) communication.^1^ Specifically, we consider two proposed explanations of the “how” of recipient design and present evidence that the computationally simpler of the two cannot by itself account for the subtle and context-sensitive ways in which humans fine tune their messages to addressees.

Traditionally, recipient design is thought to involve a mechanism that forms hypotheses about, among other things, beliefs, and knowledge of the addressee, and uses these hypotheses to optimize the message for the addressee (Grice, 1975, 1989; Clark and Carlson, 1982; Levelt, 1989). Such a *perspective taking* mechanism can explain several of the adaptations made by Ann in our example. For instance, observing the addressee's accent, Ann may infer that English is not his first language and therefore that he is unlikely to know low frequency words and understand grammatically complex English sentences. She may in turn use this (inferred) information to construct simpler sentences that she believes are understandable for the addressee.

In more recent years, researchers have argued that a perspective taking mechanism for recipient design is computationally too costly to be plausibly invoked automatically in everyday communication (Epley et al., 2004; Shintel and Keysar, 2009; Galati and Brennan, 2010). These researchers propose that instead recipient design is based on simple heuristics or rules-of-thumb triggered by the presence or absence of certain cues.^2^ Such a cue-based *heuristics* mechanism for recipient design may achieve communicative fine tuning without any resort to hypotheses about the beliefs and knowledge of the addressee. To illustrate, consider again the example scenario: Ann may take the foreign accent as a cue to classify the addressee as a tourist and the habitual response triggered by this classification may be to speak more clearly, use shorter sentences, use higher frequency words, etc. Again, as a result of such adjustments Ann may construct a message that the addressee is more likely to understand than otherwise. Observing such communicative fine tuning one may think Ann designed the message for the tourist based on what she thinks he knows and believes, but in fact this would be a case of mere appearance of perspective taking. Given the presumed intractability of recipient design by perspective taking, and the evident availability of an alternative and computationally cheaper heuristics account, it seems prudent to investigate if perhaps the computationally simpler account can by itself account for recipient design in human communication.

Understanding the computational sufficiency of different mechanisms for recipient design is also of considerable practical importance. For example, it can give us insight into how to create artificial agents that can communicate in human ways (e.g., in the context of human-robot interaction; Breazeal, 2002; Green et al., 2008). Imagine a situation where Ann is in a shopping mall and is being approached by a robot who wishes to provide her with information about an attractive sale (Shiomi et al., 2007; Satake et al., 2009). How should the robot adapt its communicative signals such that Ann will better understand it? If the adaptation could be achieved by a set of simple heuristics this could make the design of such socially interactive robots much more feasible, as compared to when the adaptation would require the robot to engage in elaborate hypothesizing about the beliefs and knowledge of the addressee.

In this paper, we investigate the computational sufficiency of simple heuristics-based mechanisms for explaining recipient design as it occurs in human–human communication. We specifically set out to identify situations in which humans adapt communicative signals in ways that cannot be explained by simple heuristics. As our examples illustrate, it can be difficult to tease apart perspective taking and heuristics in natural language conversation. For this reason, we study recipient design in the context of a communication game in which players create novel communicative signals in the absence of previous conventions. The form of communication occurring in this game can be compared to real-world situations where two agents act without a completely shared lexicon, such as when speaking to a tourist or when signaling something from a distance or behind a window. The game that we use is called the *Tacit Communication Game* (TCG, De Ruiter et al., 2010) and it has been previously validated in several studies.

## 2. Recipient design in a game context

The TCG has been developed to study human communication under controlled experimental conditions (De Ruiter et al., 2007, 2010; Newman-Norlund et al., 2009; Noordzij et al., 2009). The game is part of a general approach to the study of human communication that goes under the label of *experimental semiotics*. This approach has been contrasted by Galantucci (2009) with *experimental pragmatics*. Whereas experimental pragmatics focuses on spoken conversation, experimental semiotics is concerned with human communication more generally and the emergence of novel ways of communicating in particular (Galantucci and Garrod, 2010). Experimental semiotics is characterized by the use of games in which participants are to discover novel communicative systems. By studying communication in experimental semiotic games it becomes possible to test for fundamental characteristics of communication free from the conventions introduced by linguistic settings. Semiotic games also give more experimental control on the common ground shared by participants during communication. Several semiotic games have been developed and studied (Camerer, 2003; Weber and Camerer, 2003; Galantucci, 2005; Selten and Warglien, 2007; Scott-Phillips et al., 2009; Feiler and Camerer, 2010), with the TCG being one of the few that has been studied both from a behavioral and neuroscientific perspective.

**Figure 1:**
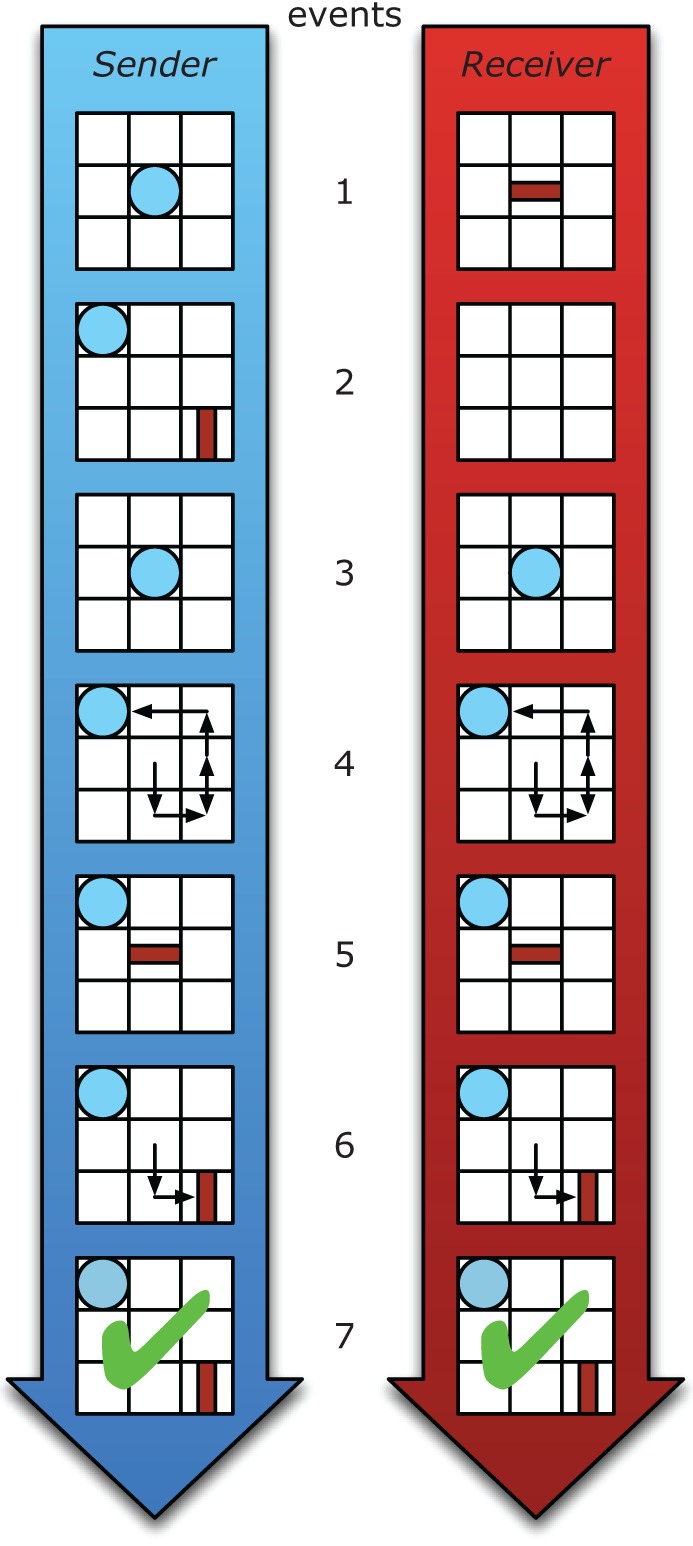
**The chronological order of phases in a trial of the Tacit Communication Game, left (in blue) is the sender and right (in red) the receiver.** In phase 1 both sender and receiver are presented with their assigned token for this trial. Next, after the sender presses a start button, in phase 2 the receiver is presented with a blank screen while the sender is shown the goal configuration of both tokens and she plans her movements (unrestricted time). After the sender presses the start button again, phases 3 and 4, both players' screens display the sender's (blue) token and the sender is able to move her token for 5 sec. It is during this phase that the sender can communicate the relevant information of the goal configuration to the receiver using movements of her token. After the sender is finished, she either presses the start button or the 5 sec time limit expires and phases 5 and 6 begin. Here both players' screens display the receivers (red) token and the receiver can move his token. Now the receiver should move his token to the location (and orientation) that he has inferred from the sender's movement. Finally, after the receiver has finished moving his token, both players receive feedback for their performance on this trial. A green check mark denotes that both players' tokens are in the exact same location and orientation as depicted in the goal configuration shown to the sender in phase 2; and a red cross (not shown in this figure) denotes that the tokens are not placed correctly.

The TCG is a communicative task where two players, a sender (referred to as *she*) and a receiver (referred to as *he*) play a game on a 3 × 3 grid board. Figure 1 depicts the sequence of events in a typical communicative trial. Here, only the sender knows a goal state that has to be reached in a cooperative fashion by her and the receiver (e.g., the circle is to end up in the upper left corner and the rectangle in the lower right corner, see event 2 in Figure 1). The senders' task is to signal the receiver what his goal is by moving her token on the board (e.g., a circle). At the same time she is to contribute to achieving the final goal state by moving her token to its goal position (e.g., the sender's circle must end up in the upper left corner of the board, but along the way signal to the receiver that he is to place his rectangle in the lower right corner). Although the TCG may look superficially very dissimilar to everyday face-to-face (linguistic) communication, in fact it is designed to capture the fundamental problems faced by human communicators during their daily interactions. For instance, in the TCG the sender gives directions to the receiver using non-conventional means on the basis of limited common ground. The structure of this communicative problem closely matches that of the scenario described in the Introduction, where a tourist asks Ann for directions. More generally, every human starts without access to the local communicative conventions. Accordingly, the TCG addresses the human ability to quickly build new semiotic conventions, while providing strong experimental control of the communicative setting, and precise quantification of the communicative behavior of the interlocutors.

Previous research has shown that in this game senders engage in recipient design, i.e., they tune their communicative signals to the particular receiver who is their current co-player. For instance, De Ruiter et al. (2010) observed that game performance (i.e., number of correct goal configurations produced by the two players) improved when senders received feedback about whether or not their signals were successful in communicating with the receiver. This finding suggests that senders use this feedback to better tune their signals to the receiver. Also, in a variant of the TCG adapted to child-level complexity, Newman-Norlund et al. (2009) observed that (adult) senders make very specific changes to their communicative signals depending on whether or not they believed to be playing with an adult or a child. For instance, they observed that initially the length of the pause by the sender's token on the receiver's goal location—taken to be an ostensive signal—was significantly longer when the sender was told the co-player was a child rather than an adult. Given that performance of the receiver was identical in the two conditions—viz., the receiver was played by an experimental confederate—the effect slowly disappeared as the sender got further tuned to the current co-player.

Findings such as these show that the TCG evokes recipient design, making the game a suitable platform for our study. Although the abovementioned findings were previously interpreted as evidence for a perspective taking mechanism for recipient design, these observations could also be explained using cue-based heuristics mechanisms. For instance, the finding that performance improves with feedback can be explained by a “take-the-best” heuristic (Gigerenzer and Goldstein, 1996), which selects signals from a predefined list based on their cue validity and where cue validity is updated on the basis of the received feedback. The finding that signals are initially different when a sender thinks she is playing with a child versus an adult, yet become comparable when performance of the co-players turns out to be identical, can be explained by an “anchor-and-adjust” heuristic (Tversky and Kahneman, 1974; Epley et al., 2004). Such a heuristic can adopt different anchors for discriminability of a signal for different categories of addressees and adjusts these discriminability values upon finding that lower levels suffice as well.

Additionally, a study by Noordzij et al. (2009) showed that the right posterior Superior Temporal Sulcus (right pSTS) is active in both senders (during planning) and receivers (during observation of the signal). Noordzij et al. reasoned that the right pSTS implements an intention recognition process that is used by receivers to understand signals, but also by senders as a subprocess of recipient design. Their finding, however, does not unequivocally show that sender's engage in this form of perspective taking. Namely, it is also consistent with the idea that the pSTS implements the shared representations of senders and receivers that are activated during communication.

These observations are not to argue that the results in the literature are not suggestive of perspective taking. We merely wish to point out that the evidence is not yet conclusive: the findings do not rule out that the effects can be explained by simple heuristic mechanisms as well. Moreover, given the prevalent idea that perspective taking is computationally costly, whereas heuristics are computationally cheap, the latter may prima facie make for a more plausible explanation of the effects than the former. By studying in more detail context-specific dependencies between receiver behaviors and sender signals in the TCG, we aim to contribute more convincing evidence that recipient design also draws on mechanisms of perspective taking.

Specifically, we set out to study adaptations made by senders to their signals on a given trial as a function of the type of error made by the receiver on a preceding trial. Our rationale for studying such trial-to-trial dependencies is the following: if a receiver makes an error in interpreting a sender's previous signal, this may cause the sender to change her signal to make it easier to understand for the receiver—i.e., recipient design. The adaptation may be achieved by invoking some form of perspective taking. For instance, observing the error made by the receiver, the sender could form hypotheses about why the receiver misunderstood certain aspects of the signal, and then use these hypotheses to make her subsequent signals easier to understand for the receiver. Alternatively, the sender may make her subsequent signals easier to understand without any recourse to perspective taking but instead by using only simple heuristics. In the latter case, however, the nature of the adaptations should be such that they can be explained by invoking some simple function mapping error cues to adaptations. We test whether or not the trial-to-trial adaptations made by senders in the TCG can be modeled by such simple heuristic rules.

## 3. Methods

We report novel analyses of behavioral data collected by Stolk et al. (2012). The aim of Stolk et al. was to study the neural correlates of human intentional communication using MEG imaging. The experiment consisted of two tasks, namely the TCG and a comparable control task without communicative dependencies. As the tasks were completely blocked in the design, we can focus on the design of the TCG task by itself. In this section we present the methods that were relevant for acquiring the behavioral data that we analyzed.

### 3.1. Participants

Fifty-two participants, students and colleagues, took part in the study. We will report analyses of the behavioral data obtained for a selection of 46 participants. Two pairs were excluded because of technical problems and one pair because performance was exceptionally poor.^3^

Participants gave informed consent according to institutional guidelines of the local ethics committee (CMO region Arnhem-Nijmegen, The Netherlands) and were either offered a financial payment or given credits toward completing a course requirement. The age of participants ranged between 18 and 40 years and all had normal or corrected-to-normal vision.

### 3.2. Materials

Participants of each pair sat behind a 19-inch monitor on which the game board (3 by 3 squares) and the tokens were displayed. Participants controlled their token with a hand-held controller. This controller contained (among others) four buttons which were positioned left, right, up, and down from one another, these corresponded with the four directions in which a token on the board could be moved. Additionally, one of the shoulder buttons on the right side of the controller was used to perform a 90° clockwise rotation of the token. Another shoulder button on the left side of the controller could be used to indicate the beginning and/or the end of a movement interval.

**Figure 2:**
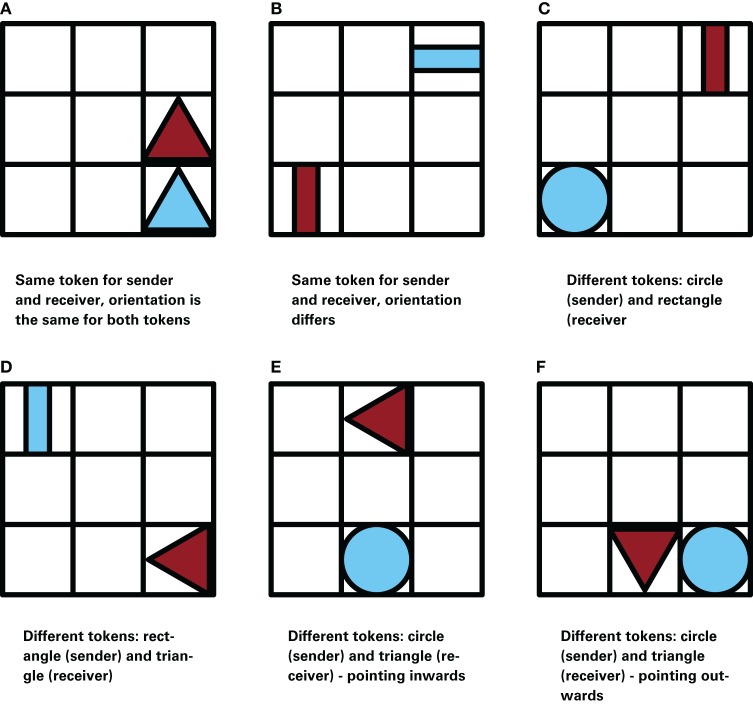
**Examples of the six different types of goal configurations.** The difficulty of a game is determined by the combinations of tokens; the boards are ordered in increasing difficulty. In these examples the sender controls the blue token while the receiver controls the red token.

In the experiment 80 goal configurations were used. We distinguish six classes of configurations of presumed different difficulty. These classes are graphically illustrated in Figure 2.

### 3.3. Procedure

Participants first read and signed an informed consent form, received standardized written instructions for playing the TCG and the control task, and of each pair one participant was prepared for the MEG measurements (in total approximately 20 min). After having been given opportunity to ask questions about the instructions pairs practiced using the controller (approximately 15 min). In both tasks a task-specific practice session of 20 min preceded the 80 recorded trials which took about 45 min, resulting in a total duration of the experiment of about 3 h.

Participants of a pair were in separate rooms when they played the game. Each pair played the TCG for the 80 goal configurations. We will refer to each such game as a *trial*. The ordering of trials was identical for all pairs of players. Trials were ordered in such a way that trials became progressively more difficult toward the end of the experiment. Table 1 lists the different configurations and their distribution over the 80 trials. The role of sender and receiver alternated every trial, such that each participant was sender in 40 trials and receiver in the other 40 trials. The order of events within a given trial of the TCG game is illustrated in Figure 1. Participants receiver no performance-based rewards other than positive and negative feedback (see Figure 1, event 7).

**Table 1:** **Overview showing the number of times that the different types of goal configurations occurred and how these were distributed over the time course of the experiment (indicated by trial number)**.

**Goal configuration (sender, receiver)**	**Example**	**Trial number**
Same shape, orientation not important	2(a)	1–4, 10, 16
Same shape, different orientation	2(b)	5–9, 17
Circle—rectangle	2(c)	11–15, 18–25
Rectangle—triangle	2(d)	26–27
Circle—triangle—pointing inwards	2(e)	28–45, 48–49, 51, 54, 58, 61–77, 80
Circle—triangle—pointing outwards	2(f)	46–47, 50, 52–53, 55–57, 59–60, 78–79

## 4. Results

**Figure 3:**
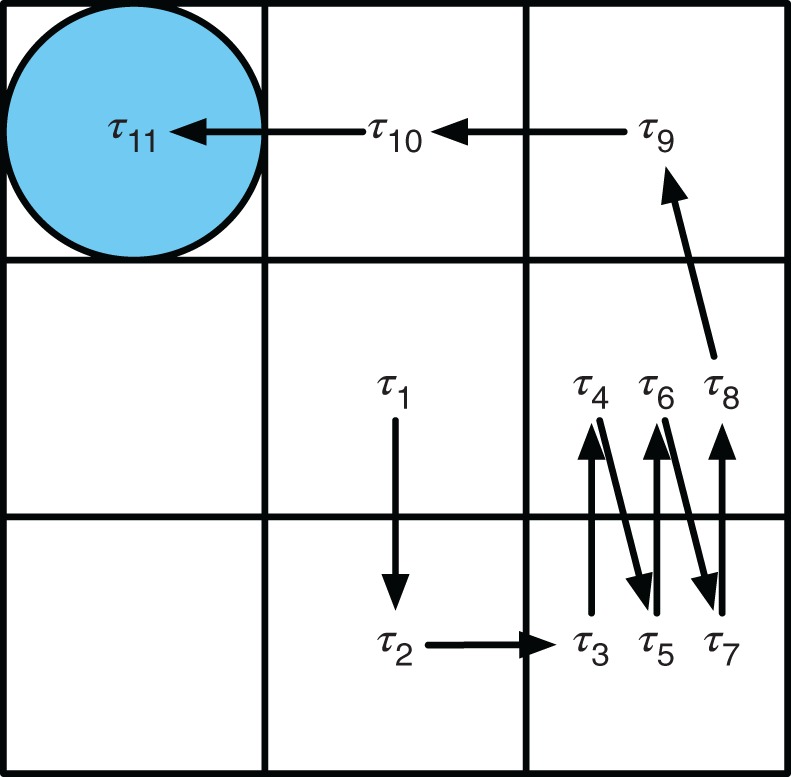
**This example movement in trial *x* illustrates how the intervals *T*^*x*^ = (τ^*x*^_1_, …, τ^*x*^_11_) that are part of a wiggle movement are divided over the three types of locations.** The goal location is the bottom-right square, the non-goal locations are the rest of the squares, and the adjacent location is the middle-right square. This means that *G*^*x*^=(τ^*x*^_3_,τ^*x*^_5_,τ^*x*^_7_), *N*^*x*^ = (τ^*x*^_2_,τ^*x*^_4_,τ^*x*^_6_,τ^*x*^_8_,τ^*x*^_9_,τ^*x*^_10_), and *W*^*x*^ =(τ^*x*^_4_,τ^*x*^_6_), as explained in sections 4.1 and 4.2.

Consistent with previous research on the TCG (De Ruiter et al., 2010), we found that senders typically develop a communication strategy in which a part of the sender's movement is designed to signal the goal *location* of the receiver's token and another (potentially overlapping) part of the movement is designed to signal the *orientation* of the receiver's token. Such compositional structure is also characteristic of everyday intentional communication. The most common strategy for communicating location is what we refer to as a *pause*, i.e., the sender's token spends relatively more time at the goal location for the receiver's token as compared to the time it spends on other squares of the board. De Ruiter et al. (2010) have previously suggested that such a pause can be seen as an ostensive signal. This pause signals its own signalhood by being dysfunctional in the sense that it deviates from the most efficient way of moving. In a similar vein, apparently dysfunctional movements were used by sender to signal the goal orientation for the receiver's token, but the variation of types of signals constructed was much larger than for signaling location (see Appendix B for an overview). The most common strategy that we observed is what we will call a *wiggle*. This strategy is illustrated in Figure 3.

Overall performance on the task (trials resulting in correctly achieved goal configurations) ranged between 31 and 75 trials correct (Mean % correct = 72%, SD = 14%). In section 4.1 we analyze adaptations made by senders to their own location signals (i.e., pauses) after receiver errors and in section 4.2, we do the same but then for senders' orientation signals (i.e., wiggles). As explained in section 4.2, we will specifically set out to test if the nature of the adaptations can be explained by simple heuristic rules.

### 4.1. Recipient design in location signals

We analyze changes to the sender's communicative signal for the receiver's location—i.e., the pause on the receiver's goal location—after three types of preceding errors on the part of the receiver (only location error, only orientation error, or both location and orientation error).^4^ To define our dependent measure we assume that the longest (most discriminable) pause on the receiver's goal location is used by the sender to communicate location to the receiver. To measure the degree to which a sender increases or decreases the relative duration (or discriminability) of the longest pause, we use a *normalized* measure of *change in duration* of pausing on the goal location. We denote this measure as Δ(*p*:*N*) and its mathematical definition is explained next.^5^

We define an ordered list *T*^*t*^ = (τ^*t*^_2_, …, τ^*t*^_*n*−1_) of intervals between individual moves (i.e., “times spent on locations”) for the entire movement of a sender's token in trial *t*, excluding the start and end intervals (i.e., τ_1_ and τ_*n*_). See Figure 3 for an illustration. We further distinguish two types of locations on the board: the receiver's goal location and the receiver's non-goal locations (i.e., the rest). The following two sublists of *T*^*t*^ contain the times that the sender's token spent on these two types of locations:

- *G*^*t*^⊆ *T*^*t*^, such that *G*^*t*^ contains all “times spent on” the goal location;
- *N*^*t*^⊆ *T*^*t*^, such that *N*^*t*^ contains all “times spent on” non-goal locations.

The length of longest pause on the receiver's goal location is defined as follows:

(1) $$p^{t}=\underset{g^{t}\in G^{t}}{\max}g^{t}$$

It is not the absolute value of *p*^*t*^ that determines the discriminability of the pause for a receiver, but how much longer *p*^*t*^ is as compared to the times spent at other locations. To capture this discriminability we normalize *p*^*t*^ with respect to the average time spent at other locations $\bar{n^{t}}$ (Equation 2). The normalized measure *p*^*t*^:*N*^*t*^ divides *p*^*t*^ by the average time spent on non-goal locations $\bar{n^{t}}$:

(2) $$\bar{n^{t}}=\frac{1}{\left|N^{t}\right|}\sum_{n^{t}\in N^{t}}n^{t}$$

(3) $$p^{t}:N^{t}=p^{t}/\bar{n^{t}}$$

Our interest is in how *p*^*t*^:*N*^*t*^ changes on trial *t* as a function of the type of error made by the receiver on trial *t*−2 (recall, sender and receiver roles switch every trial; therefore the last trial preceding trial *t* on which the sender was in the sender role is trial *t*−2). We therefore define a measure that computes the size of *p*^*t*^:*N*^*t*^ relative to the size of *p*^*t*−2^:*N*^*t*−2^. We use the log_2_-ratio as this minimizes the effect of variability in overall movement speed and allows us to treat the amount of (normalized) increase and decrease symmetrically.^6^^,^ ^7^ The resulting measure is defined as follows:

(4) $$\Delta (p:N)=\log_{2}\left(\frac{p^{t}:N^{t}}{p^{t-2}:N^{t-2}}\right)$$

We computed statistics for the measure Δ(*p*:*N*) separately for those trials where the receiver on trial *t*−2 placed his token in the incorrect location but in the correct orientation (location error), placed his token in the correct location but in the incorrect orientation (orientation error), and placed his token both in the incorrect location and incorrect orientation (combined error). In this analysis we ignore trials where on *t*−2 no receiver error seems to have been made, which would be either because the trial was successful or because the error seemed to have been due to the sender rather than the receiver. Appendix A describes in detail how we filtered those trials.

Table 2 gives an overview of the relevant statistics after removal of outliers. As the assumption of normality was violated for the three distributions of the change in pause measure, we performed a non-parametric Wilcoxon signed rank test for independent samples to test whether or not the change in normalized pause length differed from zero in the three conditions. Here values larger than 0 correspond to an increase in the length of the pause and values smaller than 0 correspond to a decrease in pause length. We found a significant increase in the length of the pause after a receiver had previously made a location error (Mean = 0.17, Median = 0.17; Percentage of trials with increased pause time = 68%, *p* < 0.04), but no significant change after an orientation error or after a combined error (*p* > 0.67 and *p* > 0.37, respectively).

**Table 2:** **Overview of results for change in pause length on trial *t* as compared to trial *t* − 2 for the three types of receiver errors**.

	***N***	**Mean**	**SD**	**Median**	***p***	**Increase (%)**
Location	37	0.17	0.47	0.17	0.04	68
Orientation	69	−0.02	0.66	−0.15	0.67	39
Location and orientation	41	0.10	0.67	0.14	0.37	56

We note that it is quite remarkable that we observe this recipient design effect after location errors despite potential variability introduced by the intervening trial (*t* − 1) on which the sender was in the receiver role. This suggests that the effect is quite robust. Of interest is whether the effect is best explained by a process of perspective taking or by the application of a simple heuristic.

At first it may seem that our finding that the pause length is significantly increased after the receiver made a location error, but not after the receiver made an orientation error, is consistent with the idea that the sender could use a simple rule such as “if location in error, then increase relative pause.” Such a rule should indeed be triggered after a location error and not when that cue is absent (i.e., when only the orientation was in error). However, such a rule should be triggered always when the relevant cue is present, yet we found no significant increase in pausing length when the receiver had made an error both in location and in orientation. It thus seems that senders interpret location errors as different kinds of misunderstandings on the part of the receiver than a combined error, causing them to highlight location after a location error, but not after a combined error. The reasons for this will become clear after our next analysis of how senders adapt their signals for orientation after receiver errors.

### 4.2. Recipient design in orientation signals

In the previous section we found a specific adaptation to the pauses—used to signal a receiver's goal location—of senders after a location error. We performed a similar investigation into the strategies used by senders to signal goal orientation to see whether or not we would observe specific adaptations to these strategies. Given that a variety of qualitatively different strategies were used by senders to signal orientation, averaging effects over these would make the results uninterpretable. Therefore, we decided to focus on the most common strategy used to signal orientation: a wiggle. Wiggle strategies were observed in trials in which sender and receiver tokens were different in shape. There were five pairs of participants that used different strategies to communicate orientation and their trials were excluded from this analysis (see Appendix A).

A wiggle is a (possibly repeated) movement of the sender's token from the receiver's goal location to one of its adjacent locations on the board, and back again to the receiver's goal location. Figure 3 illustrates this movement characteristic. The majority of pairs used the direction of this movement to communicate the orientation of a token (e.g., a wiggle toward a location above the goal location would indicate that the receiver's triangle should “point” up, toward that location). A few pairs used a different interpretation. They used the exact number of wiggles to communicate the number of rotations the receiver needed to perform in order to correctly orient his token.

We reasoned that, just like there could be a heuristic rule that states “if location in error, then pause longer”, there could be a heuristic rule that states “if orientation in error, then wiggle longer (i.e., perform more wiggles)”, or alternatively, “if orientation in error, then wiggle slower.” Inspection of sender movements revealed that although the number of wiggles performed varied between participants, it was practically constant within any given participant across all trials for those participants who used the “wiggle to point”-strategy (i.e., some participants consistently wiggled once, some consistently wiggled twice, etc.). Hence, the number of wiggles lacked the within-sender variability required for recipient design. Moreover, for participants using the “wiggle to rotate”-strategy, the number of wiggles was consistently linked to the number of required rotations. The speed of the wiggle was variable within participants and a meaningful measure for all wiggle strategies, and therefore we set out to test if it was indeed lower after orientation errors as predicted by the second hypothesized heuristic. To investigate this we defined a measure of *hange in speed* of the wiggle, which we denote by $\Delta \bar{w}$. We next explain how this measure is mathematically defined.

As before we use the list notation *T*^*t*^ = (τ^*t*^_2_, …, τ^*t*^_*n*−1_) to denote intervals between individual moves (i.e., “times spend on locations”) for the entire sender movement in trial *t*, excluding the start and end intervals (i.e., *t*_1_ and *t*_*n*_). For our purposes we consider a particular sublist of *T*^*t*^, viz., those times spent on the location adjacent to the receiver's goal location visited by the sender's token during the wiggle:

- *W*^*t*^ ⊆ *T*^*t*^, such that *W*^*t*^ contains all “times spent on” the adjacent location that were part of the wiggle.

We defined the speed of a wiggle $\bar{w^{t}}$ as the average time spent on the adjacent field as:^8^

(5) $$\bar{w^{t}}=\frac{1}{\left|W^{t}\right|}\sum_{w^{t}\in W^{t}}w^{t}$$

Naturally, the slower the wiggle, the longer the average time spent on the adjacent field is and the higher $\bar{w^{t}}$ is. We assume that the discernibility of the wiggle is independent of the movement speed of the sender. Therefore, no further normalization of Equation 5 is needed.

Our interest is in how the speed of the wiggle $\bar{w^{t}}$ changes on trial *t* as a function of the type of error made by the receiver on trial *t*−2. We define the relative change in speed of the wiggle $\Delta \bar{w}$ as:^9^^,^ ^10^

(6) $$\Delta \bar{w}=\log_{2}\left(\bar{w^{t}}/\bar{w^{t-2}}\right)$$

When $\Delta \bar{w}$ is less than 0 this means that the sender has increased the speed of the wiggle; when $\bar{w^{t}}$ is 0 the speed of the wiggle is unchanged; and when $\bar{w^{t}}$ is greater than 0 the sender would have decreased the speed of the wiggle.

We calculated statistics for the measure $\Delta \bar{w}$ separately for those trials where the receiver on trial *t*−2 made a location error, an orientation error, or a combined error. Similar to the analysis in section 4.1, we ignore trials with the property that the error on *t*−2 was not unambiguously due to the receiver (see Appendix A for details on how these trials were filtered).

Table 3 gives an overview of the relevant statistics after removal of outliers. We performed a non-parametric Wilcoxon signed rank test for independent samples to test whether or not the change in wiggle speed differed from zero in the three conditions. Here values larger than 0 correspond to an increase in the speed of the wiggle and values smaller than 0 correspond to a decrease in the speed of the wiggle. We found a significant increase in the speed of the wiggle after a receiver had previously made a combination of a location error and an orientation error (Mean = −0.13, Median = −0.10; Percentage of trials with increased wiggle speed = 24%, *p* < 0.03), but no significant change after orientation errors alone, or after location errors alone (*p* > 0.27 and *p* > 0.57, respectively).

**Table 3:** **Overview of results for change in wiggle speed on trial *t* as compared to trial *t* − 2 for the three types of receiver errors**.

	***N***	**Mean**	**SD**	**Median**	***p***	**Increase (%)**
Location	19	−0.07	0.33	−0.03	0.57	42
Orientation	39	0.04	0.20	0.01	0.27	54
Location and orientation	25	−0.13	0.26	−0.10	0.03	24

Our results do not support the type of heuristic we hypothesized for signal adaptation after orientation errors, as no change in wiggle speed was observed after those type of errors. Also, no such change was observed after a location error. Interestingly, though, if the receiver previously made an error in both location *and* orientation we did observe a change in speed, but this change was in the opposite direction than we had anticipated. That is, after a receiver had made a combination of location and orientation error on trial *t*−2 the speed of the wiggle was significantly *increased*, rather than decreased, by the sender on trial *t*. Inspecting the trials on which receivers made these combined error revealed that the increase in speed of the wiggle served a purpose in disambiguation.

**Figure 4:**
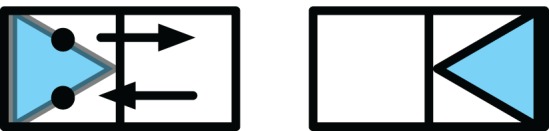
**A frequent error which occurs for the “wiggle to point”-strategy is that the wiggle and pause are confused.** In this example, the figure on the left depicts the goal configuration and the sender's movement. The figure on the right depicts the receiver's incorrect placement.

A typical error made by the receivers on these trials was to mistake the field adjacent to the goal location with the goal location itself. Conditional on this (mistaken) inference, the wiggle signaled an orientation in the opposite direction of the correct orientation, in effect causing the receiver to also incorrectly infer orientation. This situation is sketched in Figure 4. It seems that upon observing this combined error made by the receiver, the sender realizes that the misinterpretation was caused by an ambiguity in the signal making it difficult for the receiver to discern which of the two locations visited during the wiggle is the goal location. The sender then makes the discriminability between goal location and its adjacent field higher, not by increasing the relative pause on the goal location (see section 4.1), but by decreasing the (average) time spent at adjacent field. The context-sensitive nature of this adaptation of the signal for orientation (i.e., the adaptation does not occur after an orientation error, but it does occur after a combined error) suggests genuine perspective taking on the part of the sender.

## 5. Discussion

We set out to investigate whether or not recipient design in human communication can be fully explained by simple “fast and frugal” heuristics (Gigerenzer and Goldstein, 1996; Gigerenzer and Todd, 1999; Gigerenzer and Brighton, 2009; Shintel and Keysar, 2009). To this end, we studied trial-to-trial changes made by players in the context of a communication game. In this game, players had to mutually achieve a goal configuration that only one of the players knew (the sender). The sender was to communicate to her co-player (receiver) the goal location and orientation of the receiver's token by moving her token on the board. In our analyses we tested changes in movement characteristics of the sender's token movement after a receiver had made one of the following three possible errors in a preceding trial: the receiver had placed his token in an incorrect location but correct orientation (location error); the receiver had placed his token in the correct location but in an incorrect orientation (orientation error); or the receiver had placed his token in an incorrect location and incorrect orientation (combined error).

First, we found that after a receiver had made a location error, senders tended to pause relatively longer on the receiver's goal location. This change in the sender's movement can be interpreted as making the pause more discriminable from the rest of the movement, making it in effect “clearer” or “less ambiguous” which of the locations on the board was marked by the sender as the receiver's goal location. Second, we found no such increased emphasis on the goal location after an orientation error, nor after a combined location and orientation error. Particularly, the absence of an increased pause in the latter case is important, as it demonstrates that the adaptation is not guided by a simple heuristic rule such as “if location in error, then pause longer”. After all, such a rule should also be triggered when both location and orientation are in error, because its precondition would be satisfied in that case as well.

It may be argued that the pattern of data could be explained by a heuristic rule “if location in error *and* orientation *not* in error, then pause longer.” Putting aside that such a heuristic rule seems to be rather *ad hoc*, it also violates the condition of frugality that is generally taken as the hallmark of simple heuristics (Gigerenzer and Goldstein, 1996; Gigerenzer and Todd, 1999; Gigerenzer and Brighton, 2009). Namely, the extra condition “orientation is not in error” is here set to function as a context for when to apply the simple rule “if location in error then pause longer” and when not. When a heuristics program allows for this type of context sensitivity there seems to be no bound to the possible (potentially arbitrary) interactions it can code between cues and the adaptations they trigger. Mappings encoding context-sensitivity, potentially even to arbitrary levels, can hardly be said to be simple in the sense of frugal as they are not ignoring much information.

The abovementioned *ad hoc* heuristic would also not be able to account for another finding we did. After a receiver had made a combined location and orientation error, senders tended to wiggle their token relatively faster. Here, a “wiggle” was a (potentially repeated) movement of the sender's token from the receiver's goal location to an adjacent field and back to the receiver's goal location (see Figure 3 for an illustration). The wiggle movement was used by some senders to signal the “direction” of the receiver's token (the movement direction aligning with one of the main axis of the receiver's token, triangle or rectangle) and by others to signal the “number of rotations” to be performed by the receiver with his token (senders always knew the start orientation of the receiver's token). Inspection of the situations in which receivers made the combined location and orientation error revealed that it arose from a confusion on the receiver's part between the starting point of the wiggle (the goal location) and the end point of the wiggle (the location adjacent to the goal location). The confusion sometimes caused the receiver to mistakenly infer that the location adjacent to the goal location was the actual goal location; an error in orientation was then caused as a side-effect by the receiver correctly interpreting the *direction* of the wiggle movement conditioned on the erroneously inferred location (see Figure 4 for an illustration). The increase in the sender's wiggle speed can be understood as the sender disambiguating which of the locations visited during the wiggle is the goal location and which not, by spending on average less time on the location adjacent to the goal location.

Notably, we did not find any adaptation of the speed of the wiggle after a receiver had made a location error alone, nor after a receiver had made an orientation error alone. Again, particularly the absence of an increased wiggle speed in the latter case is important, as it demonstrates that the adaptation is not guided by a simple heuristic rule such as “if orientation in error, then wiggle faster.” After all, such a rule should also be triggered when only orientation is in error. Another important observation is the following: if a sender would have applied the simple “if location in error, then pause longer,” she would in effect also have disambiguated which location visited during a wiggle is the goal location. The fact that senders could have used the simple rule to achieve the same effect, but do not, suggests that they do not use such simple rules in this case at all. After all, assuming that they do use the rule when receivers make a location error, but turn it off when receivers also make an orientation error and determine some other way to achieve the disambiguation, suggests that the sender would be unnecessarily expending more than necessary cognitive resources. This is important to emphasize because one could argue that our specific experimental paradigm promotes more effortful processing than involved in typical everyday communication. Even if that were the case, the results would then suggest that communicators spend the necessary resources for engaging in perspective taking even when cognitive resources are already heavily taxed by the task and even when a heuristic would have been sufficient to achieve the same effect.

Rather than postulating *ad hoc* heuristics, we think our results are better explained by the hypothesis that the sender employs a mechanism of perspective taking. On this view, errors on the part of the receiver tell the sender something about the way in which the receiver is *(mis)interpreting* the communicative intentions driving her token movements. In effect, senders may treat a “combined location and orientation error” as an entirely different event than simply a location error *plus* an orientation error. The sender uses the errors that the receiver makes to form hypotheses about the “why” of the receiver's misinterpretations, and uses these hypotheses to adjust her movements on subsequent trials. The nature of these adaptations that we observe can be interpreted as a form of “clarification” or “disambiguation”, where the sender has realized what ambiguity had caused the receiver's earlier mistake and she adjusts the signal to ensure the same mistake is prevented from then on. The context-sensitive nature of these disambiguations suggest that they are not rote rules, but quite sophisticated forms of fine tuning. For instance, imagine the receiver had placed the token on some different location than the goal location. On a subsequent trial the sender then pauses longer on the goal location to distinguish it more clearly from all the other locations she visits. Yet, if the receiver had confused the location adjacent to the goal location visited during a wiggle for the goal location itself, then the sender wiggles faster to distinguish more clearly the goal location from the adjacent location.

In sum, the recipient design that we observe in the context of our communicative game is not straightforwardly explained by simple heuristics, yet it is parsimoniously explained by perspective taking. Of course, we cannot rule out complex heuristics for recipient design. However, a heuristics account that allows for arbitrary interactions between cues runs into the same computational intractability problem that motivated the critique of a perspective taking mechanism for recipient design in the first place. Namely, the number of possible combinations of cues grows exponentially in the number of possible cues. If rules can be triggered by arbitrary combinations of cues then an exponential number of rules will need to be stored. Such a heuristic model does not obviously scale to situations of real-world complexity with more than a few possible cues (cf. Gigerenzer, 2008), because an exponential number of rules either needs astronomical amounts of space to be stored, or—if the list of rules is stored in compressed form—it takes astronomical amounts of time to find the right rule to apply (cf. Newell, 2005; van Rooij et al., 2012).

Does this mean that recipient design is computationally intractable which ever way we explain it? We most certainly do not believe that. The fact that communicators in the game engage in recipient design shows that they have some efficient way of doing so. Moreover, the nature of the signal adaptations suggests that they draw upon a mechanism of perspective taking, suggesting that the communicators had some efficient way to invoke and use such a mechanism to their ends. As some of us have extensively argued elsewhere (van Rooij, 2008; Blokpoel et al., 2010), intractability of a cognitive model should be taken as an indication that the model has so far failed to specify the right set of situational constraints under which the modeled cognitive capacity is displayed. Hence, theories of communication in general, and recipient design in particular, must incorporate a set of situational constraints that allows such theories to explain how perspective taking computations—that are otherwise intractable—can be efficiently performed under the conditions in which we observe it. Specifying such constraints is best considered a long-term research program, though some promising initial theoretical results have been obtained (Blokpoel et al., 2011; van Rooij et al., 2011).

We close by reflecting on how our research may have implications for social neuroscience and social robotics, and the interaction between these fields. First of all, our findings suggest that the TCG game can be a fruitful empirical testing ground for neural theories of perspective taking. We have shown that trial-to-trial adaptations made by senders in this game seem to directly involve perspective taking mechanisms. The results of this study provide a quantitative, sensitive, and implicit index of perspective taking that can form the basis of a number of neurocognitive investigations. For instance, in contrast to traditional approaches to the study of the neural implementation of Theory of Mind (Fletcher et al., 1995; McCleery et al., 2011) the current index of perspective taking can provide a large number of independent read-outs (trials), ensuring sensitivity; and it is independent from verbal reports, avoiding to rely on linguistic performance. These characteristics make the current index of perspective taking particularly suitable for studying mentalizing abilities (and their cerebral implementation) in populations characterized by large variability in performance and limited access to meta-linguistic knowledge (e.g., children, patients with Autism Spectrum Disorders).

Second, our findings seem to clarify the nature of a considerable challenge for the design of socially interactive robotic agents interacting with humans in real-world settings. In Artificial Intelligence there are longstanding difficulties in devising computational mechanisms for context-sensitive processes (such as perspective taking) that are *computationally tractable*—i.e., that can scale from toy domains to real-world situations in terms of computational speed (Pylyshyn, 1987; Haselager, 1997; Dreyfus, 2007). Yet, the increasing use of robots and other artificial agents in daily life (e.g., in offices, care-giving institutions, shopping malls, and musea) will require at least a reasonable functional implementation of a recipient design capacity. Imagine a robot guide in a large museum or city taking a tourist on a tour that may last for an entire afternoon or day. To ensure the robot's efficacy it seems necessary that it can adapt to individual communicative characteristics of the tourist so as to avoid huge adaptations on the tourist's part. If fast and frugal heuristics would suffice for this interactive task then computational tractability would be guaranteed. However, if we are right in our suggestion that fast and frugal heuristics will not suffice to emulate the level of adaptation characteristic for human communication, then the computational complexities associated with perspective taking—or equivalent contex-sensitive processing—will have to be dealt with head-on by designers of socially interactive robots.

These observations also suggest a way in which social neuroscience and social robotics can actually directly inform each other. On the one hand, social neuroscience can inform social robotics: Given (1) the apparent need for social robots to engage in (simulations of) perspective taking in order to achieve human-level recipient design, (2) the evident ability of humans for effective and efficient recipient design recipient design during life interactions, and (3) the failing of AI so far to produce computational models of perspective taking that scale to the real world, social robotics may do well to look to social neuroscience for computational hypotheses about how the human brain implements the necessary perspective taking mechanisms. On the other hand, social robotics can also inform social neuroscience, e.g., by making the latter field (more) aware of the challenges of making computational models that can properly scale outside the toy domains studied in the lab. After all, for computational models hypothesized in social neuroscience to explain everyday human social interactions they should minimally be scalable, and hence tractable. The scalability problem known so well to researchers in AI and robotics is often not considered or even noticed in social neuroscience. This is possibly because experiments in social neuroscience are performed in the context of simple lab tasks, and hence computing the predictions made by said models for the lab setting may still be feasible. Yet, the scalability of such models can be critically tested by empirical analysis and implementing them in robots in order to test whether or not they yield similar levels of performance in situations of real-world complexity (similar to the real-time adaptive communicative actions performed by humans operating in everyday settings). In this way, social robotics can help constrain computational theories of recipient design in social neuroscience, viz., by providing scalability as a theoretical constraint. Given our finding that perspective taking may be a necessary component of recipient design in humans, an awareness of the computational complexity associated with computational models of perspective taking may be more useful in the study of communication than previously thought.

### Conflict of interest statement

The authors declare that the research was conducted in the absence of any commercial or financial relationships that could be construed as a potential conflict of interest.

## Footnotes

^1^As the focus of the debate in the literature has been on testing the extent to which people display egocentric bias when communicating (Horton and Keysar, 1996), it is important to point out that our research does not set out to directly contribute to that debate. In fact, considerations of the nature of the mechanisms underlying recipient design seem to be orthogonal to the question of the relative frequency of ecogentric bias in everyday communication.

^2^This heuristics account may take inspiration from the fast and frugal heuristic program in decision-making (Gigerenzer and Todd, 1999; Marsh, 2002; Gigerenzer and Brighton, 2009).

^3^Not only were these participants successful on just 30 out of 80 trials (37.5%), much less than the average performance (72%), but we also observed that these participants did not converge on a common (i.e., shared) strategy. Although one may argue that these participants still engaged their communicative abilities, we cannot investigate recipient design using our measures for their data, because our measures were defined on particular adaptations of particular common strategies.

^4^For consistency with our analysis for orientation signals (see section 4.2) we base the location signal analysis on the same type of trials, i.e., sender and receiver have different shaped tokens.

^5^Here the symbol : means normalized with respect to, e.g., *a*:*b* is *a* is normalized with respect to *b*.

^6^The log_2_ transforms the ratio such that it is 0 when there is no change, and the increase (Δ (*p*:*N*) < 0) and decrease (Δ (*p*:*N*) > 0) in speed are equidistant from 0, i.e., if a pause is two times shorter it has the same distance from 0 than if it was two times longer.

^7^If a sender moves from the starting position, to the receiver's goal location and then to her own goal location in three moves this measure is not calculable. Such trials are excluded from all analyses based on this measure, see Appendix A.

^8^Including the average time spent on the goal location would confound the measure because of standard pausing behavior on the receiver's goal location.

^9^Similar to Equation 4, we use a *log*_2_-ratio to preserve symmetry.

^10^If a sender does not use a wiggle this measure is not calculable. Such trials are excluded from all analyses based on this measure, see Appendix A.

## Appendix

### A. trial selection for the analyses

In this appendix we define which trials *t* were excluded from the analyses in Section 4. The motivation behind the exclusion is that we were interested to test adaptations made by senders to their signals on trial *t* after a receiver error on a previous trial *t*−2, the rationale being that this gives us a handle on recipient design by the sender as a function of type of error on the receiver's part. For this purpose we want to only include trials *t* in the analyses with the property that the error on *t*−2 can be unambiguously attributed to a misinterpretation by the receiver. After all, if the miscommunication that occurred on trial *t*−2 was due to an error made by the sender (e.g., she misremembered the goal configuration or made a mistake in the execution of her movements) then it is problematic to interpret changes made to the signal on time *t* as the result of a form of recipient design. For instance, any changes observed in trials following sender execution errors may simply be an artifact of the fact that the movement was not as planned or intended on trial *t*−2 whereas on *t* it is.

Table A1 gives an overview of the types of trials that were excluded from the analyses for this reason. In the remainder of this section we define and explain a set of criteria that we used to judge whether a signal was unintelligible.

**Table A1:** **Overview of which types of trials *t* − 2 were excluded from the analyses, based on our definition of intelligible signals**.

**Ended in goal configuration**			
**Sender**	**Receiver**	**Sender was intelligible**	**Excluded from analyses**
✓	✓	Yes	Yes
χ	χ	Yes	No
✓	χ	Yes	No
χ	✓	Yes	Yes
✓	✓	No	Yes
χ	χ	No	Yes
✓	χ	No	Yes
χ	✓	No	Yes

We list the criteria we used to judge if a sender's communicative signal was unintelligible, dependent on the type of error of the receiver. For instance, the type of error of the receiver can be a location error in which case we looked at whether the communication of location by the sender was (un)intelligible. Besides criteria for when the receiver's *location* was incorrect, we also list criteria for when *orientation* was incorrect. When both the receiver's orientation and location were incorrect we checked the criteria from both lists. To code which type of error the receiver made on trial *t* − 2, we checked if the final location and orientation of the receiver's token matched with the goal configuration.

Dependent on the type of error of the receiver, we judge an error as unintelligible (and thus a sender error) when:

- The location of the receiver's token is not correct (i.e., not on the goal location), and:
  1. There was no visit to the receiver's goal location, or;
  2. The experimenter (knowing both the goal configuration and the strategies used by the sender) could not recognize any movement characteristic signaling location.
- The orientation of the receiver's token is not correct, and:
  1. The signal is not based on any identified strategies (see De Ruiter et al., 2010 or Appendix B), or;
  2. The signal is based on a strategy that deviates from a previously mutually agreed strategy, or;
  3. There is an error in the execution of the strategy.
- The communicative signal corresponds to a different goal configuration than was presented to the sender (suggesting the sender has forgotten the goal configuration).

Note that those trials which were not excluded are not unconditionally included. For a trial *t* to be included in the analysis of pauses, means that for both trial *t*−2 and *t* the *p*^*t*^:*N*^*t*^ in Equation 3 needs to be calculable (i.e., besides a pause on the goal location, also at least one other field needs to be visited). For the analysis of wiggles, a trial *t* is included when a wiggle on trial *t*−2 and *t* is done, as required for computing $\bar{w^{t}}$ in Equation 5. To be clear, communicative success or failure on trial *t* itself was not a criterium for selection.

### B. strategies

This section illustrates the variety of strategies we observed, and their relative frequencies, using the most common trials (circle-triangle, pointing inwards; see Figure 2E and Table B1). These strategies were all used by senders to communicate orientation in addition to pausing behavior, that they used to communicate location.

**Table B1:** **An overview of the various strategies that were observed in the experiment, the number of pairs of participants using them, and the average success-rate per strategy**.

**Strategy**	**# Pairs**	**% correct**	***N***
Wiggle to point	15	70.4	615
Exit point	4	96.3	164
Wiggle to rotate	3	78	123
Exit from center	11	75.6	141

The total number of pairs is 23 (P = 23), and each strategy was observed N = P × 41 times, where 41 is the number of circle-triangle trials with inward pointing triangle (see Figure 2E).

#### Wiggle to point

A wiggle starts with the sender's token at the goal location of the receiver's token, the sender then moves to the adjacent field and then goes back to the receiver's goal location. The adjacent field is the field to which the triangle “points” (e.g., the red triangle in Figure 2A “points” up). The number of wiggles to signal orientation varied over pairs between one and four. An example of a wiggle, consisting of two repetitions, is illustrated in Figure 3.

#### Exit point

After indicating the location with a pause, the sender moves to an adjacent field and then moves to her own location. The adjacent field to which the sender exited the receiver's goal location indicates the direction in which the receiver's triangle points.

#### Wiggle to rotate

The number of wiggles starting from the receiver's goal location indicates the number of rotations of the receiver's triangle. At the start the triangle always points up and any rotation of the token is done clockwise which provides a one to one mapping from number of wiggles to number of rotations. Zero wiggles then means no rotation, one wiggle means “point right”, two wiggles means “point down”, and three wiggles means “point left”.

#### Exit from center

Senders communicate the goal orientation of the receiver by exiting their starting location in the center of the board in a particular direction. This direction indicates which way the receiver's triangle points. After this first move the sender moves to and pauses at the receiver's goal.

Some of these strategies were also observed in other trials. The “wiggle to point”- and “wiggle to rotate”-strategies, for example, were also sometimes observed in trials that required senders to communicate orientation in the trial types depicted in Figures 2B–2F. Note that not all strategies reported here were analyzed, but they were used in the trial selection process described in Appendix A.
